# A Simplified Prosthetic Implant Loading Protocol: 1-Year Clinical Follow-Up Study

**DOI:** 10.1155/2020/8832500

**Published:** 2020-08-03

**Authors:** Lorenzo Andreatta, Malin Bjursten Brailsford, Jakob Zwaan

**Affiliations:** ^1^Via Giovanni Reich 39, Torre Boldone 24020, Italy; ^2^Neoss AB, Arvid Wallgrens Backe 20, Gothenburg S-413 46, Sweden; ^3^(Studio Dentistico) Dr Jakob Zwaan, Via San Rocco 341, Calusco D'Adda 24033, Italy

## Abstract

**Purpose:**

To retrospectively investigate the one-year clinical outcome following a standardized treatment protocol for immediate loading. The protocol mandates predefined requirements for implant stability. If fulfilled, immediate loading of the implants is performed with a simplified prosthetic protocol which includes one-time impression at the time of surgery and definitive restoration within eight weeks.

**Methods:**

Twenty-five patients were treated with 48 Neoss ProActive Tapered implants. Minimum primary stability was established before subjecting the implants to immediate nonfunctional load. Definitive prostheses were delivered six to eight weeks from implant placement. Insertion torque (IT), resonance frequency analysis (RFA), intraoral radiographs, and impressions of implant positions were registered at implant placement. During clinical follow-up, RFA was measured at two, four, and six to eight weeks and six months from implant placement to monitor continued implant stability. Marginal bone level measurements were performed at implant placement, six-month, and one-year follow-up visits.

**Results:**

IT was over 40 N·cm for 46 implants. Two implants with IT <30 N·cm were both splinted to another implant with IT >50 N·cm, tightening the retention screw with low forces. No implants were lost during the observation period. Mean RFA measurements remained stable without any decrease during the initial six-month healing phase. Mean marginal bone remodeling was −0.47 ± 0.38 mm from implant placement to 1 year. No significant difference was found for marginal bone remodeling between implants placed in the healed bone or fresh extraction sockets.

**Conclusion:**

Within the limits of this study, it is concluded that using a simplified immediate loading protocol can be predictably applied to reduce the overall treatment time and the number of clinical sessions.

## 1. Introduction

Replacement of missing teeth with implant-supported prostheses has been demonstrated to be a predictable treatment option supported by more than 50 years of clinical experience and long-term clinical follow-up studies [1–3]. Long healing periods were originally advocated after tooth extraction, as well as after implant placement, in order to assure osseointegration of the implants prior to loading [4]. The development of clinical techniques, implant surface modifications, and optimization of implant macrodesigns has improved the conditions for achieving primary implant stability [5–9] and bone-to-implant contact [10–12]. These developments have allowed for shorter healing times and early/immediate loading protocols with a successful clinical outcome [13–17].

When applying immediate loading protocols, the risk of failure is higher than for conventionally loaded implants [13]. Primary implant stability is a requirement for the predictability of implant treatment with immediate loading protocols [14, 18, 19]. It is therefore crucial to know if stability has been achieved and the implant can be safely loaded. Insertion torque (IT) value and resonance frequency analysis (RFA) have been proposed to assess this stability with quantitative methods. IT correlates with the surrounding bone density and thickness [20], as well as factors such as the implant's macrodesign, bone condensing, and bone cutting properties. RFA offers a noninvasive method to clinically measure the stiffness of the bone/implant/transducer complex [21, 22]. Both RFA and IT are quantifiable measures assessing different aspects of stability, axial flexibility, and rotational force, respectively. Together they provide the clinician with a better understanding of the primary stability situation of the implant. RFA measurements do not exercise significant forces and thus can be utilized to monitor implant stability over time, whereas applying reverse torque to the implant during healing risks the osseointegration of the implant and is therefore not recommended.

The aim of the present clinical study was to investigate the one-year clinical outcome of a standardized treatment protocol for immediate loading. The protocol mandates predefined requirements for implant stability. If fulfilled, immediate loading of the implants is performed with a simplified prosthetic protocol which includes one-time impression at the time of surgery and definitive restoration within 8 weeks.

## 2. Materials and Methods

This present study was designed as a retrospective clinical study conducted at two private practices, using information from patient records. The study was conducted in accordance with the World Medical Association Declaration of Helsinki.

### 2.1. Inclusion and Exclusion Criteria

Medical records of all patients treated with Neoss ProActive Tapered implants (Neoss Ltd, Harrogate, UK) between May 2016 and February 2018 were screened for eligibility. Inclusion criteria were (1) patient ≥18 years of age, (2) patient signing informed consent, (3) loss of one or several teeth in one or both jaws, (4) implant placement in healed bone as well as extraction sockets, and (5) enough implant stability (IT >30 N·cm and RFA >60 ISQ) to allow for immediate (within 48h) loading of single crowns. For partial bridges, one implant was allowed a lower IT if it was splinted to an implant that fulfilled the stability criteria, and the restoration was left out of occlusion. Exclusion criteria were (1) any condition, disease, and/or medication that precluded oral surgery in local anesthesia and (2) presence of acute intraoral infection. Presence of mild but controlled periodontitis, smoking, and bruxism were considered as risk factors but not absolute contraindications.

All included patients were treated according to a standard treatment protocol used at the study clinics. The patients were examined clinically and radiographically; intraoral radiographs, orthopantomographs, and in some cases, cone beam computed tomography (CBCT). They were all informed of the surgical and follow-up procedures and gave their written consent to the treatment and follow-up plan. All patients complied with a hygiene maintenance program.

### 2.2. Surgical Protocol

Surgery was performed under local anesthesia (Articaina Pierrel 1 : 100000, Pierrel Pharma SRL, Capua CE, Italy), and a short antibiotic therapy with 1 g amoxicillin (Zimox, Pfizer, New York, NY, USA) was administered one hour prior to surgery, as well as six and 18 hours postoperatively.

**Figure 1:**
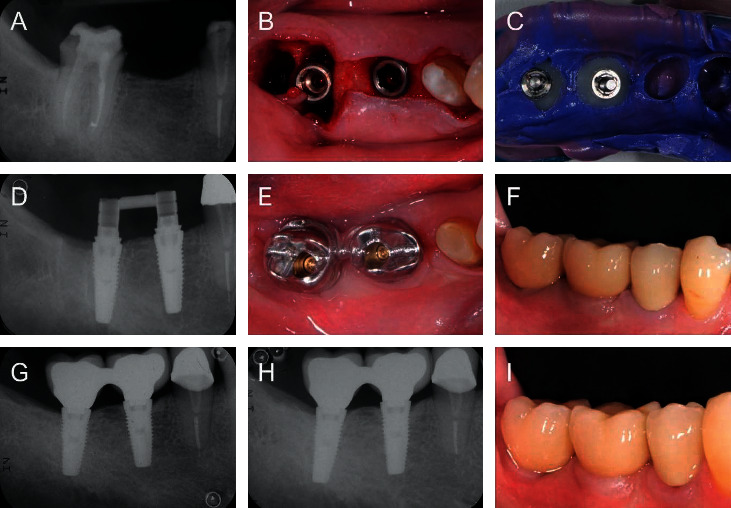
Patient subjected to partial tooth extraction and placement of two Neoss ProActive Tapered implants in the lower right molar area. (a) Presurgical situation. (b) Implant surgery. (c) Impression taking. (d) Provisional restoration. (e) Framework try-in. (f) Definitive prosthesis. (g) 6-month follow-up. (h) 1-year follow-up. (i) More than one year of clinical follow-up.

Implants (Neoss ProActive Tapered) were placed in the healed bone or extraction sockets according to the manufacturer's guidelines (Figure 1). Minor guided bone regeneration procedures were performed in cases with slight (less than 2 mm) vertical exposure of the implant at the buccal aspect. In these cases, the autogenous bone was placed directly on the implant surface, with an additional granulated bone substitute (GenOs, OsteoBiol, Torino, Italy) and no membranes. Implant stability at placement was assessed through insertion torque (IT) measurements (W&H Dentalwerk, Burmoos, Austria) as well as through resonance frequency analysis (RFA) measurements (Penguin RFA-Integration Diagnostics Sweden AB, Gothenburg, Sweden) in mesiodistal and buccolingual directions. When higher implant insertion torque was needed (IT above 50 N·cm), a manual wrench inserter was used, and IT was registered as >50 N·cm.

### 2.3. Prosthetic Protocol

A simplified prosthetic protocol was applied in which impressions of implant positions were registered at the time of implant placement (Figure 1). For both single and multiple implants, a putty and wash impression technique with resin custom spoon was performed using polyether material (Permadyne Penta L as soft material and Impregum Penta for hard material (3M ESPE, Maplewood, MN, USA)). Single implants were splinted with the resin to the spoon, and multiple implants were splinted together with the flowable composite resin and an orthodontic wire. This impression was used to fabricate the temporary as well as the definitive restoration. Screw-retained temporary restorations were made of resin-acrylic cemented onto titanium abutments (Provisional Ti Abutments, Neoss Ltd.). Temporary restorations were delivered within 48 hours after surgery. Temporary and definitive restorations were either single crowns or short-span (2-3 teeth) partial bridges on two implants. Screw-retained definitive prostheses were produced in the CAD/CAM milled Co-Cr metal (Arc Solutions, Helsingborg, Sweden) layered with feldspathic ceramic. In some cases, angulated screw-access channels were utilized to avoid unfavorable positions of the screw exit holes. At two weeks, metal framework try-in was performed, and at four weeks after surgery, clinical try-in of the definitive prosthesis was performed, checking occlusal and dynamic contacts with the 12 micron articulating paper privileging canine guidance. Papilla fill and cleansability were checked with interdental brushes to establish both esthetics and hygiene. Definitive prostheses were delivered six to eight weeks after implant placement and tightened with a handheld driver (Figure 1). Final tightening of the screws at 30 N·cm preload was done at the six-month follow-up visit, and the access holes were closed with an etching and bond technique.

### 2.4. Clinical Outcome Measures

Implant stability during the healing phase was assessed with RFA measurements (Penguin RFA-Integration Diagnostics Sweden AB) at two-, four-, and six to eight weeks and six months from surgery in mesiodistal and buccolingual directions in line with the prosthetic protocol. For these measurements, the restorations were carefully detached from the implant and remounted after complete measurement. Clinical follow-up data were collected from the six-month and one-year follow-up visits. An implant was considered as survival if it is clinically stable, complying with the function of supporting the prosthesis. Implant failure was defined as removal for any reason. Life table analysis was used to present cumulative implant survival rates (CSR). Prosthetic success was defined as absence of prosthetic complications, clinically correct fit of the metal frameworks, confirmed by radiological exams, and satisfying masticatory, esthetic, and hygienic functioning of the intact ceramic layering. Peri-implant soft tissue inflammation was examined at the six-month follow-up when the prosthesis was removed for the final RFA measurement and definitive placement. A clinical examination of mucogingival health, comprising probing the sulcus without removing the devices, was done at one year.

All adverse events recorded in the patient records were compiled.

Marginal bone level measurements were performed from intraoral periapical radiographs taken at the time of implant placement (used as radiographic baseline), six-month, and one-year follow-up visits. The upper corner of the coronal shoulder of the implant was used as the reference point. Measurements of the distance from the reference point to the first bone-to-implant contact at the mesial and distal aspects of the implant were performed, calculating a mean value.

### 2.5. Statistics

The principal outcome parameters were implant survival and marginal bone remodeling. The influence of the factors listed in Tables 1 and 2 on RFA values and bone remodeling was also evaluated. The Mann–Whitney *U* test was used to identify differences in RFA values and bone remodeling between groups. Significance level *p* < 0.05 was used for all tests. The statistical analyses were made with the SPSS Statistics 17.0 software (SPSS Inc., Chicago, IL, USA).

## 3. Results

### 3.1. Baseline Data

For this one-year retrospective study, 25 patients (11 females and 14 males, mean age 61 years) treated with 48 implants were included (Table 1). Implants were placed in the healed bone (*n* = 33) or extraction sockets (*n* = 15) according to the manufacturer's guidelines (Figure 1). Four implant sites showed slight (less than 2 mm) vertical exposure of the implant at the buccal aspect. In these cases, minor guided bone regeneration was performed. A total of 25 prosthetic constructions were made to restore the study implants: two single crowns and 23 fixed partial bridges (2-3 teeth units) (Table 2).

### 3.2. Insertion Torque (IT)

Frequency distribution of the measured IT is shown in Table 3. All implants except two measured an IT of 40 N·cm or higher. The other two implants were splinted to another implant with an IT of 50 N·cm or higher.

### 3.3. Resonance Frequency Analysis (RFA)

**Figure 2:**
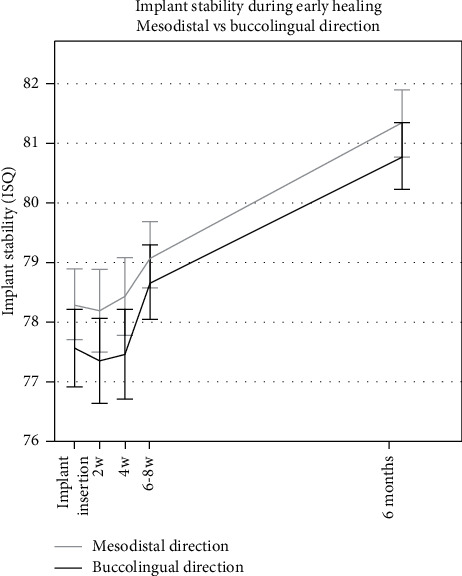
Time-stability curve for ISQ measurements taken in mesiodistal and buccolingual directions at the time of implant insertion, 2-, 4-, and 6 weeks, and 6 months. Error bars show the standard error of the mean (SEM).

**Figure 3:**
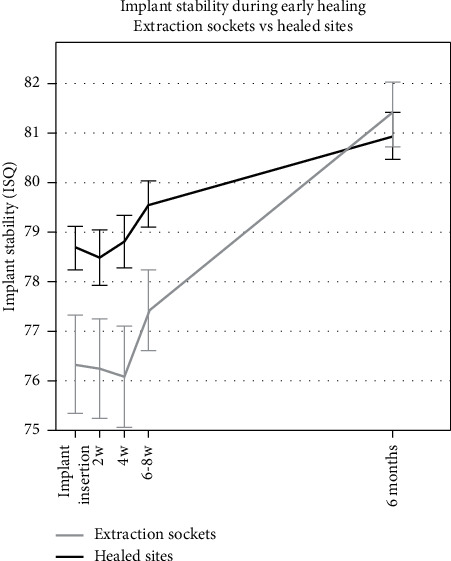
Time-stability curve for ISQ measurements comparing implants placed in healed sites and extraction sockets at the time of implant insertion, 2-, 4-, and 6 weeks, and 6 months. Error bars show the standard error of the mean (SEM).

Mean RFA measurements were ISQ 78.3 ± 4.0 (*n* = 48) in the mesiodistal direction and ISQ 77.6 ± 4.4 (*n* = 48) in the buccolingual direction at placement and ISQ 81.3 ± 3.7 (*n* = 47) and ISQ 80.8 ± 3.6 (*n* = 47) at six months, respectively (Figure 2). There was a trend showing higher initial ISQ values for implants placed in healed sites compared to those placed in extraction sockets in the early healing phase. However, the study was not designed as a comparative study, and no significant differences between the groups in either mesiodistal or buccolingual direction (Figure 3) were shown.

### 3.4. Implant/Prosthetic Survival

No implant losses occurred during the one-year observation period, rendering a cumulative implant survival rate of 100%. All prostheses met the prosthetic success criteria rendering a cumulative prosthetic success rate of 100%.

### 3.5. Radiographic Follow-Up

**Figure 4:**
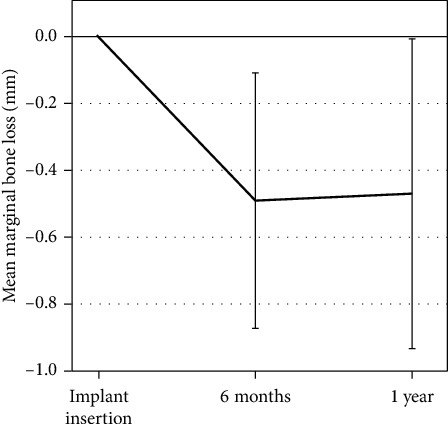
Mean marginal bone remodeling from the time of implant placement to 1-year follow-up. Error bars show standard deviation (SD).

**Figure 5:**
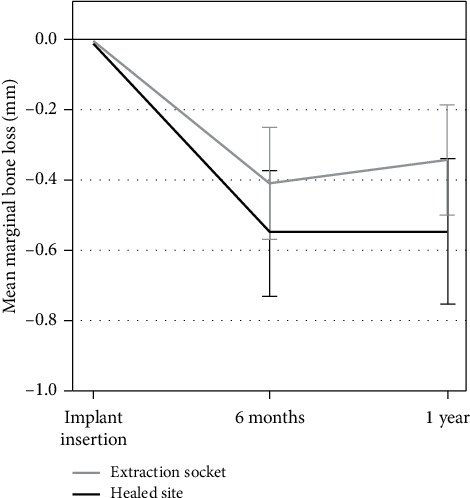
Mean marginal bone remodeling from the time of implant placement to 1-year follow-up comparing implants placed in healed sites vs extraction sockets. Error bars show the standard error of the mean (SEM).

Based on all readable radiographs, the mean marginal bone level was 0.13 ± 0.25 mm (*n* = 42) below the top of the implant collar at the time of implant placement (baseline), 0.65 ± 0.38 mm (*n* = 37) after six months, and 0.63 ± 0.46 mm (*n* = 42) after one year. Mean marginal bone remodeling was -0.47 ± 0.38 mm (*n* = 40) from implant placement to one year, −0.49 ± 0.38 mm (*n* = 35) from implant placement to six months, and +0.01 ± 0.28 mm (*n* = 37) between six and 12 months of follow-up (Figure 4). No implant sites showed more than 1.90 mm of bone resorption during the one-year observation period. There were no statistically significant differences in marginal bone remodeling between implants placed in the healed bone and those placed in fresh extraction sockets (Figure 5).

### 3.6. Clinical Follow-Up

No peri-implant soft tissue inflammation was registered during the one-year observation period. No spontaneous bleeding following crown or bridge removal for RFA measurements or other adverse events was registered.

## 4. Discussion

This 1-year retrospective clinical study, including 25 patients and 48 implants, showed that when achieving primary stability, the immediate loading protocol with early delivery of immediate prosthesis gave a predictable clinical outcome. All implants and prostheses remained in function following 1 year, giving a survival rate of 100%. This is in agreement with other 1-year follow-up studies using the Neoss implant system [9, 23]. Radiographic analysis of the peri-implant bone showed that most of the bone remodeling occurred during the first 6 months in function (−0.49 ± 0.38 mm), and stable bone remodeling occurred between the 6-month and 1-year visit (+0.01 ± 0.28 mm). Moreover, no significant differences were found for bone remodeling between implants placed in the healed bone and those placed in fresh extraction sockets. Previous findings with the Neoss implant system also reported on minimal bone remodeling for the first year in function [9, 23] as well as for longer follow-up periods [24, 25].

In the current study, only implants with an ISQ value higher than 60 at insertion were included for immediate load. RFA has been extensively documented as a tool for assessing implant stability [26, 27]. Andersson et al. recently showed a correlation between a high ISQ value and long-term implant survival [28]. Some evidence suggests that decreasing ISQ values over time may indicate higher risk of implant failure [28–31]. In the present study, implant stability was continuously monitored during the crucial initial healing phase to be able to unload implants to avoid implant failure if a decreasing degree of stability was encountered. The mean RFA measurements remained stable with only minimal changes during the initial healing phase, hence no unloading was necessary. No implant recorded an ISQ value below 66 or showed a clinically significant decrease during the initial six months of healing, indicating safe loading conditions in the early osseointegration phase for all implants.

In the current study, all single, nonsplinted implants needed to reach a minimum IT of 30 N·cm to be subjected to immediate load. This is in line with earlier studies [32] showing good clinical results for implants with high insertion torques as well as a Cochrane systematic review concluding that high IT is one of the prerequisites for a successful immediate/early loading procedure [33]. Implants with lower insertion torque were only allowed if they were splinted to another stable implant. Two implants did not reach an IT of 30 N·cm but were both part of a three-element provisional bridge which made splinting, and hence inclusion in the study population was possible. Low insertion torque is a known risk factor in implant treatment, and the splinting was a prerequisite for treatment. One implant showed a high insertion torque during placement and exactly when reaching its final seating spun. Bone quality and bone-implant contact were judged to be excellent, and the implant did not rotate when forced with a handheld driver. The second implant was placed with Summer's technique using an osteotome that was slightly overdimensioned, thus widening by compression and not by removing bone from the implant site. Bone-implant contact at the implant platform level was complete. Special attention was dedicated to assure that the provisional had no contact in occlusion and that lateral forces were minimized during dynamic function. The retention screws were finger-tightened (<10 N·cm) in order to minimize rotational forces on the implant and thereby mitigate retention loss from multiple removal of the temporary restorations during the first eight weeks of treatment. Both implants with low IT remained in function throughout the study period. Their stable ISQ values during healing and minimal marginal bone remodeling indicate success also for these low IT implants. This is in line with the study by Norton et al. which showed that immediate provisionalization of single-tooth implants placed with a relatively low IT (≤25 N·cm) could yield favorable survival rates with optimal maintenance of marginal bone levels [34].

All implants in the study were subjected to immediate load where delivery of the final prosthesis occurred within eight weeks from implant placement. Obtained by one-time impressions, only one set of casts were used for delivery of the provisional acrylic restoration and the definitive ceramic restoration. This simplified prosthetic loading protocol has many benefits for both the patient and the dentist: (1) shorter treatment times with fewer surgical operational interventions, thus less discomfort for the patient, (2) only one impression at the time of surgery, and (3) immediate loading (within 48 hours). When the temporary prosthesis is placed during the very first stages of healing, before the blood clot has been reorganized, the soft tissue will easily adapt to the designed profile. Gingival epithelium can migrate on smooth and clean surfaces and thus will be prone to follow the shapes as defined in the dental laboratory [35]. During the healing phase, the clinician can check gingival levels and soft tissue compression and if necessary adapt the emergence profiles by grinding or adding material. These chairside modifications can be copied to the master CAD file by scanning the temporary prosthesis mounted on the master model and merge the two CAD files, creating a new master file that contains the modified emergence profiles.

The biggest limitation of the current study is its design as a retrospective study with a limited sample size and no control group. However, despite their limitations, retrospective single-cohort studies provide valuable clinical data adding to the overall clinical evidence of the documented procedure.

## 5. Conclusion

This study presents how a simplified immediate loading protocol, when taking into account implant stability by means of RFA and IT, can achieve a predictable clinical outcome with reduced overall treatment time and number of clinical sessions.

## Figures and Tables

**Table 1:** Patient demographics.

Parameter	Group	*n*	%
Age (61.1 ± 11.2)	30–39	1	4.0
	40–49	4	16.0
	50–59	2	8.0
	60–69	12	48.0
	70–79	5	20.0
	80	1	4.0

Gender	Female	11	44.0
	Male	14	56.0

Smoking	No	22	88.0
	Yes	3	12.0

Bruxism	No	19	76.0
	Yes	6	24.0

**Table 2:** Baseline implant/site data.

Parameter	Group	*N*	%
Implant position	Anterior maxilla	2	4.2
	Posterior maxilla	20	41.7
	Anterior mandible	1	2.1
	Posterior mandible	25	52.1

Jaw	Maxilla	22	45.8
	Mandible	26	54.2

Implant length	7 mm	—	—
	9 mm	4	8.3
	11 mm	25	52.1
	13 mm	17	35.4
	15 mm	2	4.2

Implant diameter	3.5 mm	5	10.4
	4.0 mm	24	50.0
	4.5 mm	15	31.3
	5.0 mm	3	6.3
	5.5 mm	1	2.1

Bone quality	A1	2	4.2
	A2	33	68.8
	A3	13	27.1

Type of site	Healed	33	68.8
	Extraction	15	31.2

Perio	No	40	83.3
	Moderate	8	16.7

Placement	Subcrestal	17	35.4
	Equicrestal	31	64.6

Indication	Single	2	4.2
	Partial	46	95.8

Bone grafting	No	44	91.7
	Yes	4	8.3

Defect	No	34	70.8
	Yes	14	29.2

**Table 3:** Insertion torque distribution.

Parameter	Group (N·cm)	*n*	%
Insertion torque	10	1	2.1
	20	1	2.1
	40	2	4.2
	50	4	8.3
	>50	40	83.3

## Data Availability

Due to the ethical and legal responsibility to respect participants' rights to privacy and to protect their identity, the clinical dataset is not made publicly available.
